# Hibernoma: a rare benign soft tissue tumour resembling liposarcoma

**DOI:** 10.1259/bjrcr.20170067

**Published:** 2018-02-13

**Authors:** Tom Kovitwanichkanont, Parm Naidoo, Pedro Guio-Aguilar, James Leong

**Affiliations:** 1 Department of Rheumatology, Monash Health, Melbourne, VIC, Australia; 2 Department of Dermatology, Monash Health, Melbourne, VIC, Australia; 3 University of Sydney, Sydney, NSW, Australia; 4 Department of Radiology, Monash Health, Melbourne, VIC, Australia; 5 Monash University, Melbourne, VIC, Australia; 6 University of Melbourne, Melbourne, VIC, Australia; 7 Department of Plastic Surgery and Reconstructive Surgery, Monash Health, Melbourne, VIC, Australia

## Abstract

Hibernoma is a rare benign soft tissue tumour that can mimic a liposarcoma on radiographic imaging. Our case series review illustrates the clinical presentation and radiographic appearances of four patients with histologically confirmed hibernoma. Hibernoma is usually hypointense relative to subcutaneous fat on *T*_1_ weighted MRI and demonstrates partial fat suppression on fat-saturated sequences. Large intratumoral vessels likely support the diagnosis of hibernoma but are not invariably present. Fludeoxyglucose avidity on PET scan is not beneficial in distinguishing hibernoma from soft tissue malignancy because of its inherent, metabolically active property. Owing to the radiographic heterogeneity of hibernoma, it is currently not possible to diagnose hibernoma based on imaging characteristics alone. Given the excellent prognosis of hibernoma with marginal excision alone, an appreciation of the radiographic features is helpful in the appropriate pre-operative workup of soft tissue tumours.

## CASE 1

**Figure 1.**
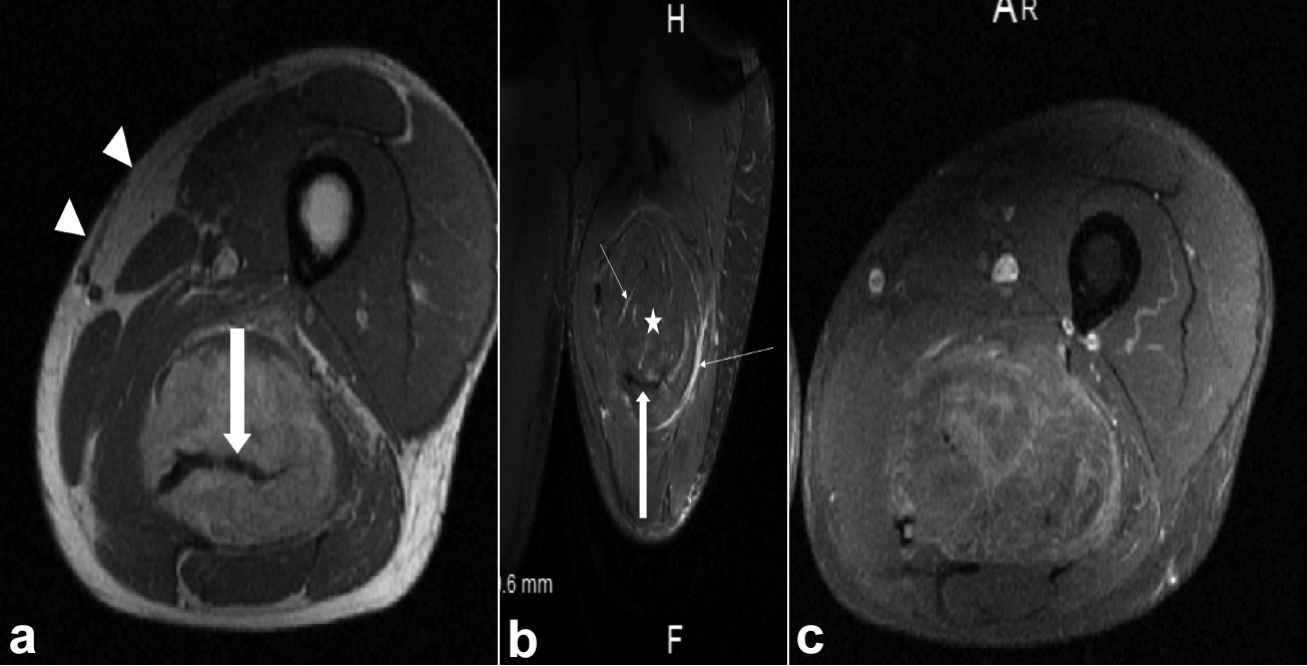
(a) Axial *T*_1_ MR image of the left upper thigh demonstrating a well-circumscribed, encapsulated large *T*_1_ hyperintense intramuscular mass, with a large flow void (thick arrow). Note that the *T*_1_ signal of the mass is less than that of subcutaneous fat (arrow heads). (b) Coronal *T*_2_ Fat-suppressed image of the left thigh demonstrating incomplete fat suppression of the lesion (asterisk, relative to subcutaneous fat) and *T*_2_ hyperintense septations and draining veins (thin arrows). Note hypointense arterial flow void (thick arrow). (c) Axial post-gadolinium fat suppressed *T*_1_ weighted MR image of the lesion, with heterogeneous enhancement.

**Figure 2.**
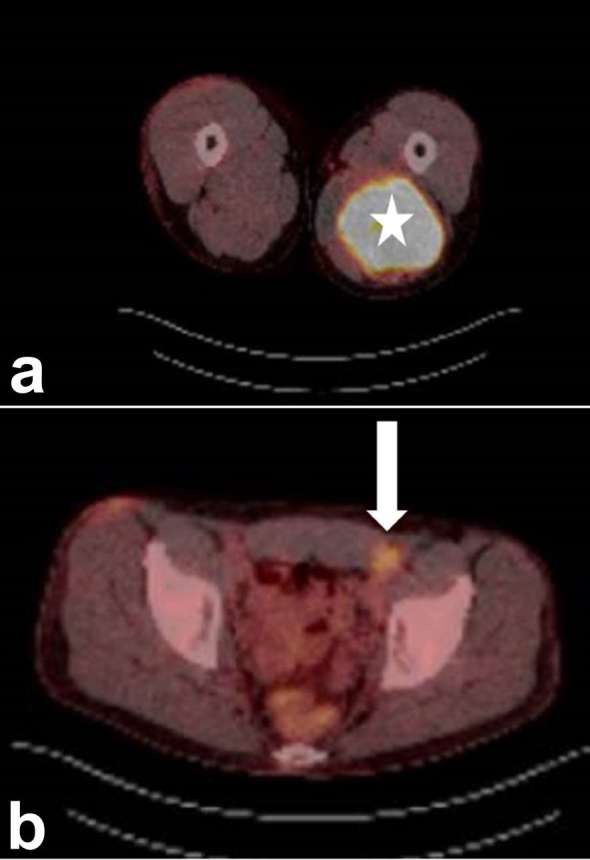
FDG PET/CT demonstrating intense radiotracer uptake (a) in the left thigh lesion and (b) in an adjacent inguinal lymph node (arrow). FDG, fludeoxyglucose.

A 21-year-old male was admitted for a pre-operative work-up of a tensor fascia latae perforator-free flap to a defect over the left ankle following a motorbike accident. During the clinical examination, he was noted to have an incidental finding of a warm mass over the adductor musculature of the left thigh. On further questioning, the patient complained of 2 months of mild pain over the thigh mass. Subsequently, an MRI of the left thigh showed findings that were reported as a low grade intramuscular liposarcoma (Figure 1a–c). A combined fludeoxyglucose PET/CT scan was performed for staging of a possible liposarcoma, which demonstrated intense FDG avidity throughout the left thigh mass [maximum standardized update value (SUV-max) of 18.4], along with several mildly avid and enlarged left groin (SUV-max of 3.3) and external iliac nodes (SUV-max of 3.8) (Figure 2a,b).

Following a discussion at a multidisciplinary meeting, patient underwent a CT-guided core biopsy of the most FDG avid part of the left thigh mass and an ultrasound-guided left inguinal lymph node core biopsy. The thigh core biopsies were suggestive of hibernoma; the histology showed brown fat composed of adipocytes with vacuolated and granular cytoplasm and small, round, bland nuclei. No cytological atypia was identified. The left inguinal lymph node biopsies revealed features suggestive of only reactive changes, with no evidence of neoplasia.

**Figure 3.**
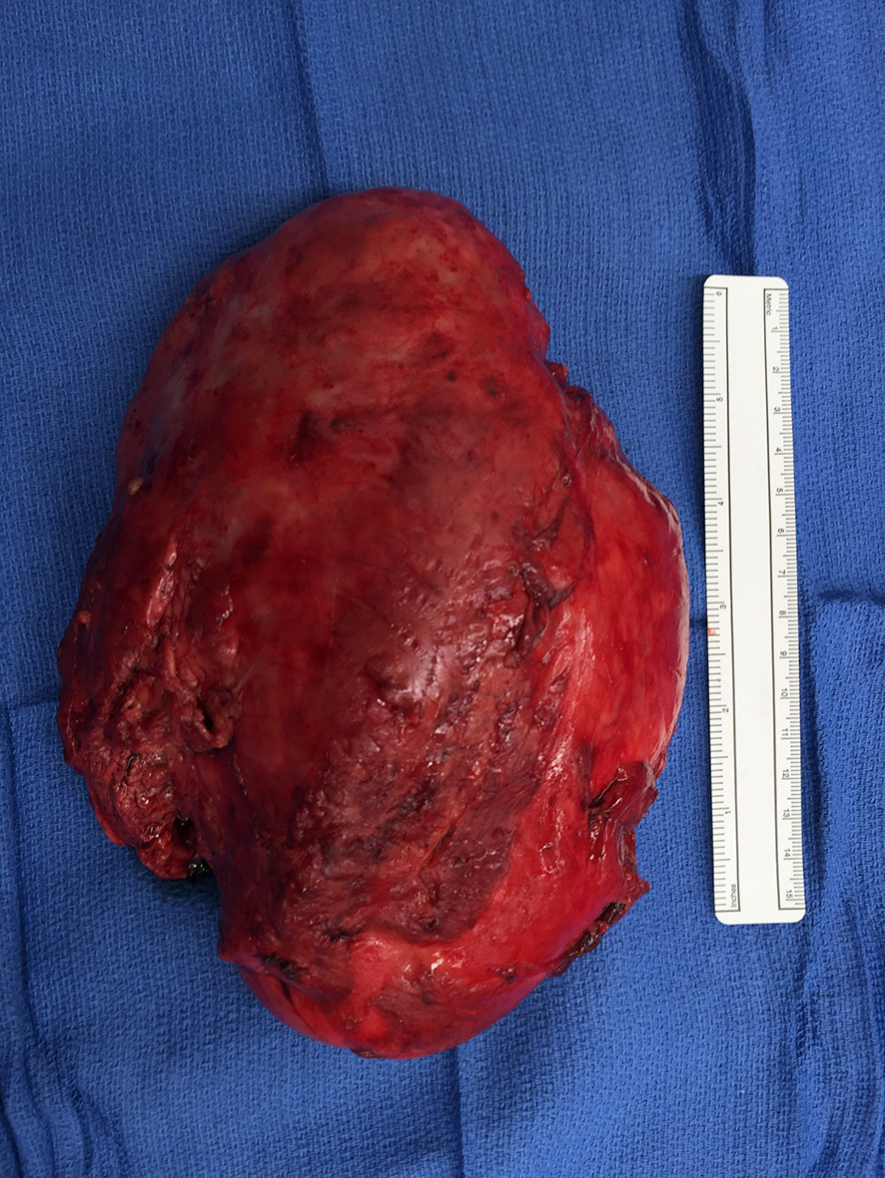
Surgical specimen of the excised left thigh hibernoma.

The patient subsequently underwent an elective wide local excision of the left thigh mass. He had an uneventful post-operative course and was discharged from the hospital on Day 5. On clinic reviews, the patient had no complaints and was able to return back to work with full function and good cosmesis of the treated leg. The histopathology confirmed an intramuscular hibernoma. The excised mass measured 17 × 13 × 6.5 cm and weighed 800 g (Figure 3). The vascularity of the lesion was more marked than the typical lipoma with relatively thick walled arteries extending through the lesion. The lesion appeared adequately excised. Owing to the benign nature of the mass and no reported cases of recurrence after complete excision, no adjuvant therapy was arranged.

## CASE 2

**Figure 4.**
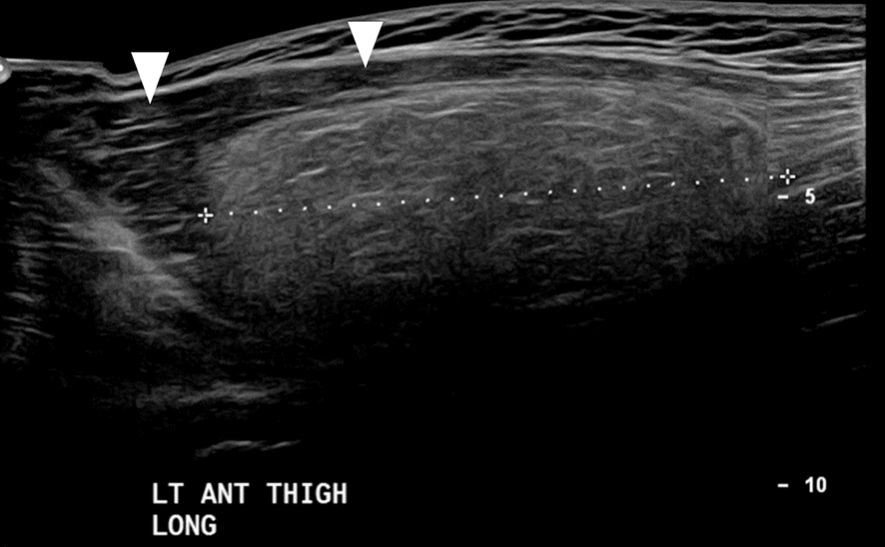
Long axis ultrasound scan of the left thigh demonstrating a large well-circumscribed intramuscular heterogeneous lesion (measuring cursors), which is hyperechoic to muscle (arrow heads).

**Figure 5.**
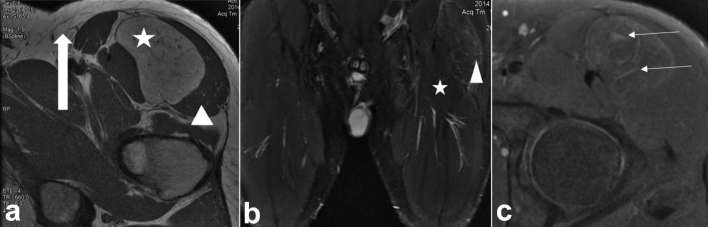
(a) Axial coronal *T*_1_ weighted image demonstrating the well-marginated, entirely intramuscular mass (asterisk), which is hyperintense to skeletal muscle (arrow head) but slightly hypointense to subcutaneous fat (thick arrow). Note low signal fine septations and arterial flow voids. (b) Coronal fat-suppressed *T*_2_ weighted image shows the lesion (arrow head) to be slightly hyperintense relative to skeletal muscle (asterisk). (c) Axial post-gadolinium fat-suppressed *T*_1_ weighted image of the upper thigh in the same patient shows mild generalized enhancement of the mass with more pronounced enhancement of the internal septations (thin arrows).

A 26-year-old male was referred for the management of a large lump on his left anterolateral thigh. The patient noticed the lump after the area was traumatized, whilst playing rugby. An ultrasound scan was performed as an initial investigation showing a non-specific intramuscular lesion (Figure 4). An MRI scan of the left thigh revealed a well-circumscribed lobulated mass that measured 6.6 × 4.5 × 11.3 cm in the anterior compartment of the upper thigh, deep to tensor fascia lata (Figure 5a–c). The imaging features were again suspicious for liposarcoma. A core biopsy was subsequently performed, which showed small tissue fragments comprised of mature adipocytes and multivacuolated brown fat cells with small nucleoli. However, the sample cell was inadequate for definitive assessment of a soft tissue neoplasm. The left thigh mass was later confirmed by an incisional biopsy to be hibernoma and was totally excised. Patient made a full recovery.

## CASE 3

**Figure 6.**
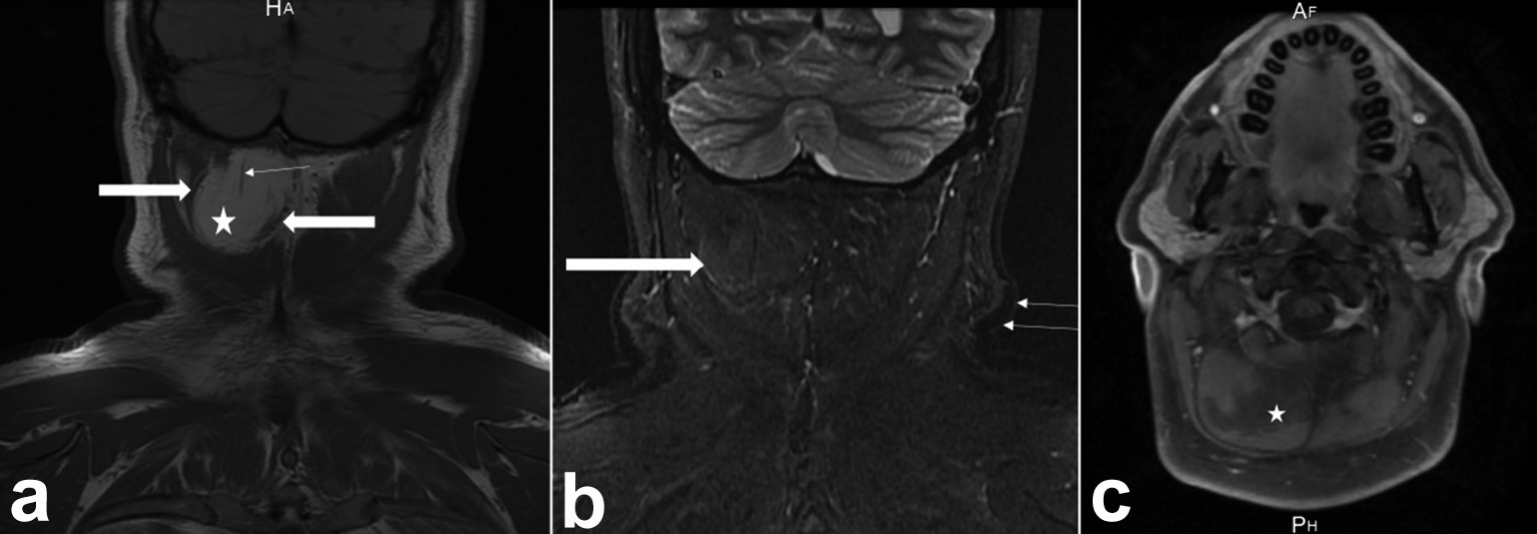
(a) Coronal *T*_1_ weighted MR image of the neck demonstrating a mass (asterisk) with signal similar to subcutaneous fat within the right semispinalis capitus muscle. (thick arrows) Note hypointense septations (thin arrows). (b) Coronal STIR MR image in the same patient demonstrating the intramuscular lesion with slightly higher signal (thick arrows) than subcutaneous fat (thin arrows) after application of fat suppression. (c) Axial post-gadolinium fat suppressed *T*_1_ MR image demonstrating no enhancement of the right-sided intramuscular lesion (asterisk). STIR, short tauinversion-recovery.

A 53-year-old male presented following an incidental finding of a mass on an MRI scan of the neck. The lesion was asymptomatic and there were no palpable masses on physical examination. An MRI scan of the neck revealed a 2.5 × 3.8 × 4.2 cm lobulated mass within the right semispinalis capitis muscle (Figure 6a–c). An excision was performed of the irregular, moderately firm, encapsulated fatty mass. Microscopically, there was lobulated adipose tissue containing numerous areas of brown fat with small foci of admixed skeletal muscle, consistent with a hibernoma.

## CASE 4

**Figure 7.**
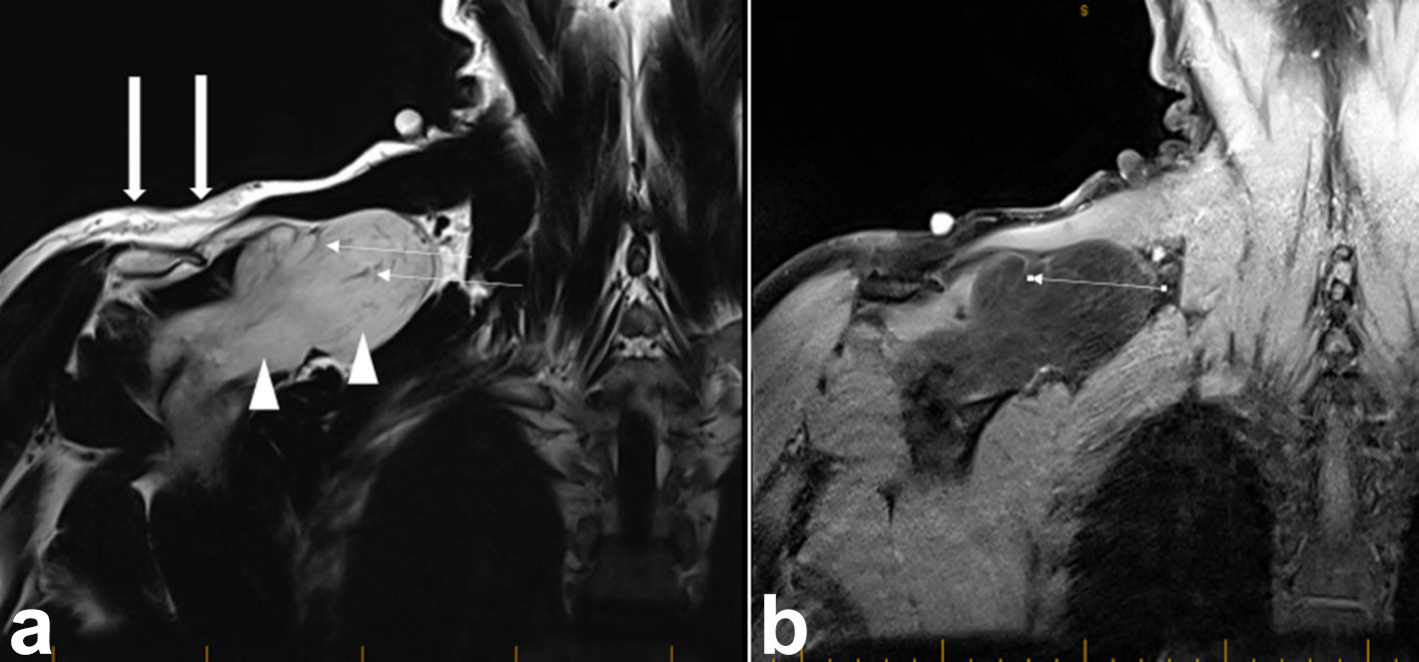
(a) Coronal *T*_1_ MR image of the right shoulder girdle demonstrating the encapsulated well-circumscribed, lobular lesion (arrow heads), with signal slightly hypointense to subcutaneous fat (thick arrows). Note *T*_1_ hypointense septations (thin arrows). (b) Coronal post-gadolinium fat suppressed *T*_1_ MRI demonstrating mild generalized enhancement of the majority of the lesion and more intense enhancement of the septations (thin arrow).

A 52-year-old male presented with an asymptomatic right shoulder mass for investigations. MRI revealed a well-circumscribed mass that measured 6.0 × 7.2 × 8 cm (Figure 7a,b). An incisional biopsy revealed the diagnosis of hibernoma. Subsequently, the mass was surgically resected and was confirmed histologically to be hibernoma. Intraoperatively, there was evidence of hibernoma extending into the glenoid fossa. The patient was discussed at a multidisciplinary meeting. A decision was made to not pursue with further arthroscopic removal of residual tumours. The team elected to monitor with surveillance MRI imaging given the benign nature of the condition. Post-operatively, the patient had a good range of motion of the right shoulder and recovered well.

## DISCUSSION

Hibernoma is a rare benign soft tissue tumour that consists of brown fat, immature fat cells. The term hibernoma was first described in 1914, owing to its resemblance to the brown fat in hibernating animals, where large quantities were found.^1^ The primary function of these fat cells is thermogenesis. Brown fat is also physiologically present in non-hibernating species, including newborn human, but it typically regresses with age comprising less than 0.1% of total body weight by the age of 70 years.^2^ Hibernomas arise from the vestiges of fetal brown fat; according to the largest series published to date, the thigh was the most commonly affected site.^3^ Other common sites include neck, back, axilla and shoulder. Hibernomas are most common in the third or fourth decades of life.^3^

They are generally asymptomatic, slow-growing and warm to touch owing to its hypervascularity. However, they can sometimes be associated with pain and weight loss. In our case series, pain was reported in only the first case owing to its significant size and the subsequent mass effect. It has been postulated that weight loss is attributed to the hypermetabolism of brown fat, but further studies are required to elucidate the underlying pathophysiology. It is recommended^4^ that patients should be made aware of the potential effect of weight gain following a removal of a large hibernoma. Owing to the insidious onset of hibernoma, the true incidence is unknown as most lesions often go undiagnosed.

Previous reports discourage preoperative core needle biopsy in suspected cases of hibernoma owing to the potential risk of bleeding resulting from its hypervascularity.^5, 6^ In our case series, preoperative core needle biopsies and incisional biopsy were performed without any bleeding complications. Hibernomas are benign tumours and complete excision results in cure in all reported cases. Recurrence has not been reported in the literature, except in a case where complete excision was not possible owing to its location impinging on the brachial plexus and axillary vessels.^7^

The imaging features of hibernomas are unfortunately, largely non-specific. Table 1 provides a summary of the key radiographic findings in our case series. Plain radiography is of no value, other than to suggest the presence of a hyperlucent (fatty) lesion, and exclude adjacent bone involvement. Ultrasound usually reveals a hyperechoic mass,^8^ which is consistent with our finding in Case 2. However, ultrasound is also of limited value, as it is generally unable to characterize tissue components accurately, and is limited in its ability to assess the full depth and extent of the lesion. CT performs better in terms of assessing the extent of the lesion, the presence of fat and hypervascularity, manifesting as visible vessels, and enhancement of parts of the lesion after intravenous iodine contrast administration.

**Table 1.** MRI and FDG PET radiographic findings of hibernoma

**Patient number**	**Age/** **gender**	**Size**	**Location**	**MRI *T*_1_ weighted signal**	**Fat suppression**	**Gadolinium contrast enhancement**	**Internal structure**	**FDG PET SUV-max**
1	21M	17 × 13 × 6.5 cm	Thigh	Hyperintense to muscle hypointense to subcutaneous fat	Incomplete	Yes	Internal septation large flow voids	18.4
2	26M	6.6 × 4.5 × 11.3 cm	Thigh	Hyperintense to muscle hypointense to subcutaneous fat	Incomplete	Yes	Internal septation small flow voids	N/A
3	53M	2.5 × 3.8 × 4.2 cm	Neck	Hyperintense to muscle isointense to subcutaneous fat	Incomplete	No	Internal septation	N/A
4	52M	6 × 7.2 × 8 cm	Right shoulder	Hyperintense to muscle slightly hypointense to subcutaneous fat	Incomplete	Yes	Internal septation	N/A

FDG, fludeoxyglucose; N/A, not applicable; SUV-max, maximum standardizeduptake value.

MRI is the imaging modality of choice, as it provides superior characterization of tissue types and greater delineation of the margins of the lesion. In general, hibernomas are well-circumscribed, encapsulated masses, most frequently seen in areas where there is a preponderance of brown fat, such as thigh, shoulder, back, neck and mediastinum.^3^ These lesions are rarely seen in the retroperitoneum, despite brown fat being commonly found in the peripancreatic and suprarenal retroperitoneum.^9^ On *T*_1_ weighted imaging, all four cases demonstrated hyperintensity compared to skeletal muscle, but hypointense relative to subcutaneous fat in three cases, and isointense to fat in one patient. These findings are concordant with the literature.^10, 11^ Incomplete fat suppression, as in all our cases, precludes the diagnosis of a simple lipoma. On fat-suppressed MRI images, signal suppression in a hibernoma may be incomplete because of the nature and amount of the lipid content.^9^ After gadolinium contrast, enhancement is variable but generally present and heterogeneous.^10^ In our patients, three lesions showed significant gadolinium enhancement, but one lesion showed negligible enhancement. Large flow voids indicating intratumoral vessels with fast flowing blood are common and offer some degree of specificity for the diagnosis of hibernomas as opposed to angiolipomas, haemangiomas, liposarcomas and other soft tissue sarcomas.^12^ The latter lesions typically lack large intratumoral blood vessels.^2, 12,13^ In our patient with a massive 800-g hibernoma, large flow voids were detected, which strongly supported the pre-operative diagnosis of hibernoma. Furthermore, there is evidence to suggest that internal septation is one of the features of hibernoma.^14^ Internal septations were present in all our cases. The heterogeneous imaging appearances of hibernomas, including the frequent demonstration of non-uniform fat signal on MRI means that a firm diagnosis of this entity is very seldom made on MRI prior to biopsy, liposarcoma being the main imaging diagnosis requiring consideration.

There is overlap in imaging features among fat-containing lesions, such as hibernomas, myolipomas, lipomas and liposarcomas. The overlap is made more complicated with the variable degree of differentiation of liposarcomas. For example, internal septations are seen in most of hibernomas but also in lipomas and liposarcomas. The thickness (>2 mm) and nodularity of internal septations has been suggested as a possible feature favouring liposarcomas over less aggressive lesions. However, fine septations, which are seen in hibernomas and lipomas, are non-specific and can still be seen in well-differentiated liposarcomas.^15^

Another differential diagnosis for hibernomas is the myxoid type of liposarcomas. This entity, which is more common in lower extremities, typically shows low *T*_1_ and *T*_2_ signal on MRI, which helps in differentiations from hibernomas. They can also demonstrate cystic changes, which are typically not evident in hibernomas.^16^ Another type of soft tissue sarcoma is clear cell sarcoma, which can exhibit intense enhancement similar to hibernomas. However, the signal characteristics of *T*_1_ intensity being similar or slightly higher than muscle intensity should differentiate this entity from hibernomas.^17^

Fluoro-labelled 2-deoxyglucose PET scan (FDG-PET) is commonly utilized as a diagnostic imaging tool to detect metabolically active tumours based on their FDG uptake. It has been proven that brown fat has a high level of FDG uptake.^18^ Brown fat cell expresses unique mitochondrial uncoupling protein (UCP1), which functions to generate heat instead of adenosine triphosphate production.^19^ A landmark study based on 3640 consecutive FDG PET and PET/CT scans demonstrated that the biopsies of the FDG-avid regions have the histological and molecular characteristics of brown fat remnants, including UCP1. The lack of FDG enhancement helps in differentiating lipomas from other lesions, such as hibernomas, liposarcomas and myolipomas. Hibernomas show increased FDG avidity more than expected for other lipomatous lesions, which can suggest the diagnosis.^20^ In general, an FDG-PET SUV in excess of 2.0–2.5 is considered concerning for malignancy. As demonstrated in our first case with SUV-max 18.4, FDG-PET lacks specificity as FDG uptake occurs in any regions of high utilization of glucose regardless of the underlying pathology, including benign, malignant or inflammatory processes. This extremely high level of FDG-PET avidity may be considered even excessive for sarcomas and may be suggestive of hypermetabolic brown fat, *i.e.* hibernoma. Hoshi et al^21^ and Charest et al^22^ concluded that FDG PET/CT lacks the specificity required to differentiate benign from malignant soft tissue tumours. In addition, FDG-PET may not be sufficiently sensitive in detecting myxoid liposarcoma owing to the limitation in discerning metabolically active cells within the myxoid matrix.^23^ The value of FDG-PET in soft tissue tumours may lie in its accuracy in discriminating low- and high-grade sarcomas.^22^ If an FDG-PET imaging is obtained, we recommend that hibernomas should be considered prospectively in the work-up of lipomatous tumours with high SUV levels, since an excision without adjuvant therapy is curative owing to their benign nature. Previous studies demonstrated that a single dose of oral propranolol (a non-selective beta-blocker) can reduce FDG avidity in brown fat, since brown fat contains beta-adrenergic receptors.^24–26^ In the future, we plan to investigate whether oral propranolol administered prior to FDG-PET can be used to help differentiate hibernoma from soft tissue malignancy.

## LEARNING POINTS

1. Hibernoma is a rare, slow-growing, benign tumour that demonstrates characteristic but non-specific imaging features. MRI is the superior imaging modality of choice, compared to plain radiography, ultrasonography and CT. If present, large arterial flow voids within a *T*_1_ hyperintense lesion are suggestive of the diagnosis of hibernoma.
2. FDG avidity offers poor specificity in the discrimination between soft tissue malignancy and benign lesions. Hibernoma should be included in the differential diagnoses of an FDG avid lipomatous tumour.
3. Owing to the current lack of definitive imaging modality for diagnosing hibernoma, a histopathological confirmation is required to exclude soft tissue malignancy.
4. Marginal excision of hibernoma offers excellent prognosis.
